# Glucagon-like peptide-1 (GLP-1) receptor activation dilates cerebral arterioles, increases cerebral blood flow, and mediates remote (pre)conditioning neuroprotection against ischaemic stroke

**DOI:** 10.1007/s00395-021-00873-9

**Published:** 2021-05-03

**Authors:** Shereen Nizari, Marina Basalay, Philippa Chapman, Nils Korte, Alla Korsak, Isabel N. Christie, Shefeeq M. Theparambil, Sean M. Davidson, Frank Reimann, Stefan Trapp, Derek M. Yellon, Alexander V. Gourine

**Affiliations:** 1 Centre for Cardiovascular and Metabolic Neuroscience, Neuroscience, Physiology and Pharmacology, University College London, Gower Street, London, WC1E 6BT UK; 2 The Hatter Cardiovascular Institute, University College London, London, WC1E 6HX UK; 3 Wellcome Trust/MRC Institute of Metabolic Science, University of Cambridge, Cambridge, UK

**Keywords:** Brain arterioles, Brain capillaries, Cerebral blood flow, Glucagon-like peptide-1, Ischaemic stroke, Middle cerebral artery occlusion, Neuroprotection, Remote ischaemic preconditioning

## Abstract

Stroke remains one of the most common causes of death and disability worldwide. Several preclinical studies demonstrated that the brain can be effectively protected against ischaemic stroke by two seemingly distinct treatments: remote ischaemic conditioning (RIC), involving cycles of ischaemia/reperfusion applied to a peripheral organ or tissue, or by systemic administration of glucagon-like-peptide-1 (GLP-1) receptor (GLP-1R) agonists. The mechanisms underlying RIC- and GLP-1-induced neuroprotection are not completely understood. In this study, we tested the hypothesis that GLP-1 mediates neuroprotection induced by RIC and investigated the effect of GLP-1R activation on cerebral blood vessels, as a potential mechanism of GLP-1-induced protection against ischaemic stroke. A rat model of ischaemic stroke (90 min of middle cerebral artery occlusion followed by 24-h reperfusion) was used. RIC was induced by 4 cycles of 5 min left hind limb ischaemia interleaved with 5-min reperfusion periods. RIC markedly (by ~ 80%) reduced the cerebral infarct size and improved the neurological score. The neuroprotection established by RIC was abolished by systemic blockade of GLP-1R with a specific antagonist Exendin(9–39). In the cerebral cortex of GLP-1R reporter mice, ~ 70% of cortical arterioles displayed GLP-1R expression. In acute brain slices of the rat cerebral cortex, activation of GLP-1R with an agonist Exendin-4 had a strong dilatory effect on cortical arterioles and effectively reversed arteriolar constrictions induced by metabolite lactate or oxygen and glucose deprivation, as an ex vivo model of ischaemic stroke. In anaesthetised rats, Exendin-4 induced lasting increases in brain tissue PO_2_, indicative of increased cerebral blood flow. These results demonstrate that neuroprotection against ischaemic stroke established by remote ischaemic conditioning is mediated by a mechanism involving GLP-1R signalling. Potent dilatory effect of GLP-1R activation on cortical arterioles suggests that the neuroprotection in this model is mediated via modulation of cerebral blood flow and improved brain perfusion.

## Introduction

Stroke remains one of the most common causes of death and disability worldwide [42]. Interventions to restore cerebral blood flow to the viable tissue surrounding the infarct core, called the penumbra, are efficient if applied within hours after the stroke onset and limited to a large artery clot removal by intravenous thrombolysis or invasive mechanical thrombectomy [74]. Many smaller vessels, however, remain constricted and contribute to the no-reflow phenomenon after stroke. This highlights the need for the development of novel therapies that can effectively improve/restore the microvascular flow and salvage viable brain tissue from permanent ischaemic damage [4].

Our body is capable of recruiting powerful innate mechanisms of inter-organ protection against ischaemia/reperfusion injury. These mechanisms effectively protect the heart and the brain and can be activated by cycles of ischaemia/reperfusion applied to an organ or tissue remote to the organ being protected. This well-characterized phenomenon is called remote ischaemic conditioning (RIC) [23, 31, 33, 39, 47]. More than 40 preclinical studies demonstrated that RIC can protect the brain against ischaemic stroke [14, 49, 72, 84]. Results of clinical studies [37] demonstrated that RIC improves brain perfusion, as well as visual-spatial and executive functions in patients affected by cerebral small vessel disease [83]. However, the mechanisms of RIC-induced neuroprotection are not fully understood and have been proposed to include reduction of astrogliosis, maintenance of blood–brain barrier integrity, the opening of K_ATP_ channels, prevention of let-7a and miR-43 overexpression, and other mechanisms [15, 20, 48, 69, 70, 77, 82].

Previous studies on the mechanisms of RIC-induced cardioprotection against ischaemia/reperfusion injury suggested that RIC recruits interacting neuronal and humoral mechanisms [8, 24, 26, 52, 75]. Our studies showed that these include sensory (afferent) innervation of the peripheral tissue undergoing RIC, autonomic parasympathetic (vagal) pathways, and the actions of an incretin hormone glucagon-like-peptide-1 (GLP-1), as a likely humoral mediator of RIC [5, 6, 62, 63]. We hypothesised, therefore, that GLP-1 may also mediate RIC-induced neuroprotection against ischaemic stroke.

GLP-1 is an important hormone that has multiple functions from the regulation of insulin secretion to the control of satiety and modulation of autonomic nervous system activity [2, 34–36]. GLP-1 is released by the enteroendocrine cells in the gut and also by the groups of specialized CNS neurons located in the brainstem [35, 66]. Experimental studies conducted in gerbils, mice and rats demonstrated that GLP-1 receptor (GLP-1R) agonists Exendin 4 (Ex4), liraglutide or semaglutide, administered either systemically or centrally, are highly effective in protecting the brain against ischaemic stroke induced by bilateral carotid artery occlusion or middle cerebral artery occlusion [9, 21, 50, 59, 78]. In at-risk diabetic patients, semaglutide was shown to significantly decrease the incidences of non-fatal strokes [60, 61]. However, the mechanisms underlying GLP-1R-mediated neuroprotection remain largely unknown [19, 59].

In this study, we tested the hypothesis that GLP-1 mediates neuroprotection induced by RIC. First, we determined the effect of GLP-1R blockade on RIC-induced neuroprotection, and then investigated the expression of GLP-1R and the effects of GLP-1R activation on cerebral blood vessels and brain blood flow, as a potential mechanism of GLP-1R-mediated neuroprotection against ischaemic stroke.

## Methods

All animal experiments were performed in accordance with the European Commission Directive 2010/63/EU (European Convention for the Protection of Vertebrate Animals used for Experimental and Other Scientific Purposes) and the UK Home Office (Scientific Procedures) Act (1986) with project approval from the University College London Institutional Animal Care and Use Committee. The study was designed and performed, the data analysed and reported in accord with the ARRIVE guidelines. Animals were maintained on a 12-h light/dark cycle and had ad libitum access to food and water.

### Models of ischaemic stroke and remote ischaemic conditioning in rats

The experiments were conducted in male Sprague–Dawley rats (220-250 g) (*n* = 39). The animals underwent surgery to allow transient middle cerebral artery occlusion (MCAO) to induce cerebral ischaemia, as an experimental model of stroke [76]. Rats were anaesthetised with isoflurane (4% induction, 2% maintenance) and the right common carotid artery was exposed. A size-matched silicon-coated monofilament (Doccol Corporation) was advanced through the right common and internal carotid arteries towards the middle cerebral artery junction until the resistance was met (distance ~ 2 cm). The wound was closed by removable stitches, analgesia was administered (Vetergesic, 0.01 mg kg^−1^, i.p.), and the animals were placed in a heated recovery chamber. The animals were allowed to recover from anaesthesia to determine the presence of the functional signs of cortical ischaemia, including walking towards the contralateral side, left forelimb flexion and body rotation to the left when the animal was held by the tail. Animals not displaying these signs were excluded from the study. After 75 min of MCAO, the animals were re-anaesthetised with isoflurane and placed on a servo-controlled heating pad (body temperature maintained at 37 ± 0.5 °C). After 80 min of MCAO, sham-RIC or RIC was applied. The wound was re-opened to expose the carotid arteries, the MCA occluder was withdrawn 90 min after the onset of ischaemia, the common carotid artery was ligated, and the wound was closed by suturing. The animals were placed in a recovery chamber until their complete recovery, before returning to their home cages.

**Fig. 1:**
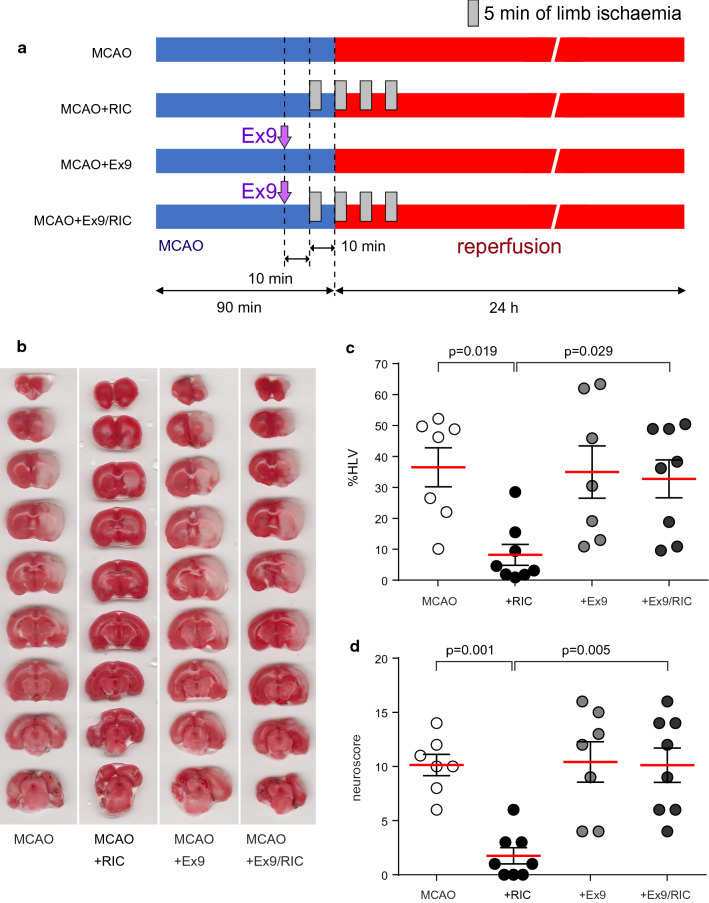
GLP-1 receptors mediate neuroprotection against ischaemic stroke established by remote ischaemic conditioning. **a** Illustration of the experimental protocols. In rats, ischaemic stroke was induced by 90 min of middle cerebral artery occlusion (MCAO) followed by 24 h of reperfusion. Remote ischaemic conditioning (RIC) was established following 4 cycles of 5-min left hind limb ischaemia interleaved by 5-min reperfusion periods. Arrows indicate the time (20 min before the onset of reperfusion and/or 10 min prior to the application of the first RIC cycle) of intravenous administration of GLP-1 receptor antagonist Exendin(9–39) (Ex9). **b** Representative images of TTC-stained coronal brain sections from four experimental groups 24 h after MCAO. **c**, **d** Summary data illustrating the effect of RIC on brain infarct size and behavioural neurological traits 24 h after MCAO. Neuroprotection induced by RIC was prevented by systemic GLP-1 receptor blockade with Ex9. Infarct size is presented as a percentage of the hemispheric lesion volume (HLV). MCAO group, *n* = 7; MCAO + RIC group, *n* = 8; MCAO + Ex9 group, *n* = 7; and MCAO + Ex9/RIC group, *n* = 8 rats. Individual data and means ± SEM are shown. *p* values—ANOVA followed by Sidak’s correction for multiple comparisons

The animals were randomized into four experimental groups: (1) rats with MCAO/sham-RIC; (2) rats subjected to RIC starting 10 min before the onset of reperfusion; RIC protocol involved 4 cycles of 5 min left hind limb ischaemia interleaved with 5-min reperfusion periods [10, 29], applied using an inflatable 12-mm cuff (IVM, USA). The cuff was inflated to 200 mmHg to stop the blood flow through the limb, as reported previously [12]; (3) rats with MCAO treated with a competitive GLP-1R antagonist Exendin (9–39) (Ex9, 50 μg kg^−1^, intravenously) 20 min before the onset of reperfusion; (4) rats subjected to the RIC protocol starting 10 min before the onset of reperfusion and treated with Ex9 10 min prior to the first episode of RIC. Experimental timeline is illustrated by Fig. 1a.

Twenty four hours after MCAO, all animals underwent behavioural neurological assessment using the 0–22 scale, as described in detail previously [9, 79]. The principal points of the functional status evaluation were spontaneous activity, gait, postural signs, lateral resistance, limb placing, and parachute reflex. Higher neuroscores indicated more severe neurological deficits. The rats were then euthanized with sodium pentobarbital overdose (200 mg kg^−1^, i.p.). The brains were immediately removed, sectioned at 1.25-mm thickness, stained with 1% triphenyl tetrazolium chloride (TTC) and fixed in formalin. The sections were photographed, and the infarct areas were determined by computerized planimetry (Image J). Infarct sizes (IS) are presented as percentages of the hemispheric volume (%HLV). Functional status evaluation and IS measurements were performed by the investigator blinded to the experimental animal group allocation.

### Acute brain slice preparation

Young Sprague–Dawley rats (P21 of either sex) were humanely killed by cervical dislocation, the brains were removed and placed in ice-cold artificial cerebrospinal fluid (aCSF) containing 124 mM NaCl, 26 mM NaHCO_3_, 3 mM KCl, 2 mM CaCl_2_, 1.25 mM NaH_2_PO_4_, 11 mM MgSO_4_, 10 mM glucose saturated with 95% O_2_/5% CO_2_ (pH 7.4). Coronal cortical slices (thickness 300 µm) were cut using a vibratome and then incubated at room temperature for 1 h in a standard aCSF solution containing 1 mM Mg^2+^ saturated with 95% O_2_/5% CO_2_.

### Recordings of cerebrovascular responses

Recordings were made from slices placed on an elevated grid in a flow chamber at ~ 32 °C (flow 2 ml min^−1^). Cortical arterioles and capillaries were identified by the diameter of the vessel (arterioles > 10 µM; capillaries < 10 µM) and the presence of smooth muscle, as described [64]. Brightfield imaging recordings of cerebrovascular responses induced by experimental treatments were performed using a Zeiss Axioskop 2 upright microscope with a 40 × water immersion objective and a Hamamatsu CCD camera. Images were acquired every 30 s and vessel diameter changes were measured using ImageJ. The percentage change in vessel diameter from the baseline was calculated and the last 5 min of vessel diameter recordings from each group was averaged for comparison. Lactate (5 mM) was added to the perfusate to constrict the vessels. Lactate was used as a constricting agent because the release of this metabolite markedly increases in hypoxic and ischaemic conditions [43, 44, 57]. The model of brain ischaemia was induced by oxygen/glucose deprivation (OGD). OGD was induced by exposing the slices to aCSF saturated with 95% N_2_/5% CO_2_ gas mixture containing 7 mM sucrose, replacing glucose. The rate of aCSF flow through the chamber was increased to 4 ml min^−1^ (to reduce the time for gas exchange between the media and the atmosphere) and the period of OGD lasted for 25 min. Exendin-4 (Ex4; 100 nM, Tocris) was used as GLP-1R agonist. Ex9 (1 µM, Cohesion Biosciences) was used to block GLP-1Rs. *N*(gamma)-nitro-l-arginine methyl ester (l-NAME; 100 μM, Sigma) was used to inhibit nitric oxide synthesis. Adenylate cyclase was blocked with SQ22536 (100 μM, Tocris).

### Measurements of brain tissue PO_2_ in vivo

Young male rats (200–250 g; *n* = 11) were anaesthetised with urethane (initial dose, 1.3 g kg^−1^, i.p.; then 10–25 mg kg^−1^ h^−1^, i.v.). Adequate anaesthesia was ensured by maintaining stable levels of arterial blood pressure and heart rate, showing lack of responses to a paw pinch. The body temperature was maintained at 37 ± 0.5 °C. The femoral artery and vein were cannulated for measurement of the arterial blood pressure and administration of anaesthetic, respectively. The trachea was cannulated, and the animal was ventilated with room air using a positive pressure ventilator with a tidal volume of ∼1 ml 100 g^−1^ and a ventilator frequency similar to the resting respiratory rate (∼60 strokes min^−1^). The animal was then placed in a stereotaxic frame and a small craniotomy (~ 1 mm in diameter) was made in the parietal bone. Brain tissue PO_2_ was recorded in the left cerebral cortex using optical probes (250 μm tip diameter; OxyLite system; Oxford Optronix) placed ~ 2 mm below the surface of the brain, as described previously [58]. The operation of the sensor is based on fluorescence technology that allows real-time recordings of tissue PO_2_. In urethane-anaesthetised rats, changes in brain tissue PO_2_ parallel changes in cerebral blood flow [18], and were used in this study as a robust proxy measure of brain perfusion.

### Immunohistochemistry

Mice expressing fluorescently tagged GLP-1R (Glp1r-cre/tdRFP) [16, 71] were used to determine the expression of the receptor by the brain blood vessels. The animals were terminally anaesthetised with sodium pentobarbital overdose (200 mg kg^−1^; i.p.) and perfused through the heart with 0.01 M phosphate-buffered saline. The brains were removed and fixed overnight in 4% paraformaldehyde. Free-floating sections (20 µm thickness) were incubated (overnight at 4 °C) with anti-DsRed antibody (1:500) (Takara Bio) to detect GLP-1R expressing cells and anti-smooth muscle actin (SMA) antibody conjugated to FITC (1:350) (Sigma) for the detection of arteries and arterioles. Incubation with anti-rabbit AlexaFluor 568 (1:200, 2 h) was performed on the next day, followed by endothelial cell labelling using biotinylated lectin (1:250, 30 min) (Vector Biolabs), visualised using AMCA streptavidin (1:100, 1 h) (Vector Biolabs). Tiled images of coronal cortical cross-sections were obtained using Zeiss LSM 800 confocal microscope.

### Data analysis

Differences between the experimental groups were analysed using GraphPad Prism 6 software. Comparisons were made using analysis of variance (ANOVA) followed by Sidak’s *p *value correction for multiple comparisons or Student’s *t* test, as appropriate. Data are reported as individual values and means ± standard error of the means (SEM).

## Results

In rats, MCAO lasting 90 min led to the development of cerebral infarcts averaging 36.5 ± 6.3% (*n* = 7) of the hemisphere volume (Fig. 1b). RIC induced by 4 episodes of unilateral femoral ischaemia/reperfusion markedly reduced the cerebral infarct size (8.2 ± 3.4%; *p* = 0.019) (Fig. 1b) and improved the neurological score (from 10 to 2 on the 22-point scale; *p* = 0.001) (Fig. 1c). The neuroprotective effect of RIC was abolished by systemic GLP-1R blockade with Ex9 (Fig. 1b, c). In this experimental group (MCAO + Ex9 + RIC) the cerebral infarct size was 32.8 ± 6.1% (*p* > 0.9 vs 36.5 ± 6.3% in the MCAO + sham-RIC group) and the neuroscore was 10 ± 2 (*p* > 0.9 vs 10 ± 1 in the MCAO + sham-RIC group). Ex9 had no effect on the infarct size and behavioural neurological traits in animals subjected to MCAO and sham-RIC procedure (Fig. 1b, c). These data suggested that signalling via GLP-1Rs is critically important for RIC-induced neuroprotection against ischaemic stroke.

**Fig. 2:**
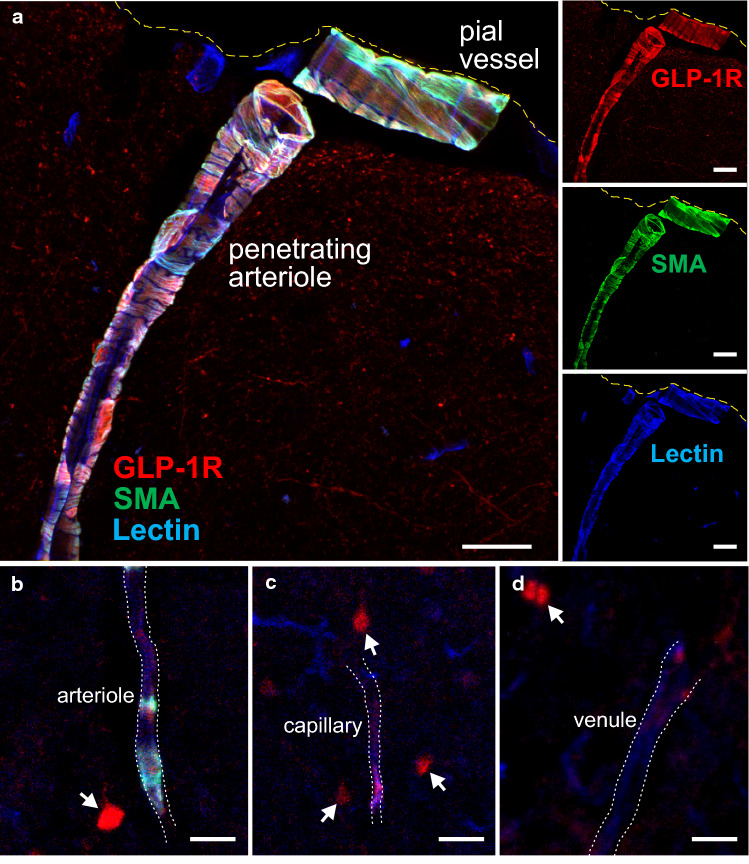
GLP-1 receptor expression in cortical blood vessels. Representative images of coronal sections of the cerebral cortex of glp1r-Cre/ROSA26-tdRFP mouse, immunostained for red fluorescent protein (red), illustrating GLP-1 receptor expression associated with penetrating cortical arteriole (**a**), parenchymal arteriole (**b**), capillary (**c**), and venule (**d**). Arteriole smooth muscle cells were labelled by immunohistochemical detection of smooth muscle actin (SMA, green). Endothelial cells were labelled with lectin (blue). Arrows point to other cortical GLP-1 receptor-positive cells. Scale bars = 20 µm

GLP-1 may act on brain vessels and aid neuroprotection by improving/maintaining cerebral blood flow in the regions surrounding the affected brain area. To test this hypothesis, we next determined the expression of GLP-1R by the brain vasculature and investigated the effects of GLP-1R activation on cerebral arterioles and capillaries. In the cerebral cortex of the GLP-1R reporter mice, expression of GLP-1R was found to be largely confined to arteriolar smooth muscle and endothelial cells (Fig. 2a, b). Patchy GLP-1R expression was detected in cells associated with isolated capillaries and veins (Fig. 2c, d). It was found that in the mouse brain 69 ± 17% of cortical arterioles, 2 ± 0.2% of capillaries and 6 ± 6% of venules express the GLP-1R.

**Fig. 3:**
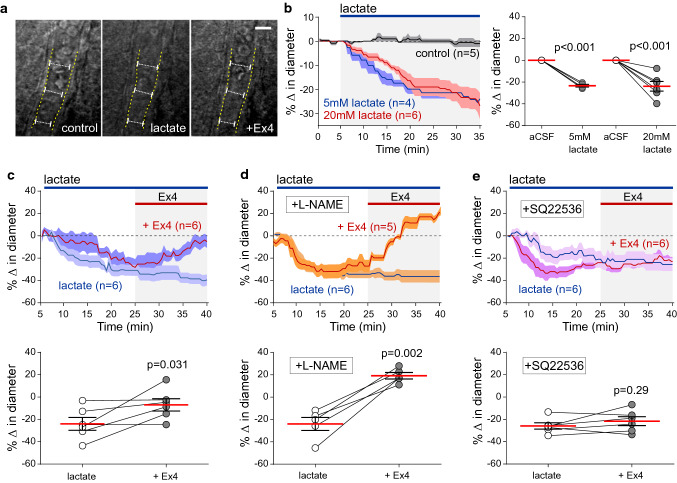
GLP-1 receptor activation dilates cortical arterioles. **a** Representative DIC images illustrating a cortical arteriole response to 5 mM lactate and GLP-1 receptor agonist Exendin-4 (Ex4; 100 nM), applied in the presence of lactate, recorded in a coronal slice of a rat cerebral cortex. The yellow dashed line outlines the edge of the vessel; the length of the white dotted line illustrates the smallest diameter of the vessel recorded during the course of the experiment. Scale bar = 10 µm. **b** Summary data illustrating changes in the diameter of cortical arterioles recorded over time and peak arteriolar responses induced by lactate applied in concentrations of 5 or 20 mM. **c** Summary data illustrating the effect of Ex4 on the diameter of cortical arterioles pre-constricted with lactate (5 mM). Lactate-evoked arteriolar constrictions were effectively reversed by Ex4. **d** Summary data illustrating the effect of Ex4 on the diameter of cortical arterioles pre-constricted with lactate (5 mM) in the presence of nitric oxide synthase inhibitor l-NAME (100 µM). l-NAME had no effect on Ex4-induced arteriolar dilations. **e**, Summary data illustrating the effect of Ex4 on the diameter of cortical arterioles pre-constricted with lactate (5 mM) in the presence of adenylate cyclase inhibitor SQ22536 (100 µM). SQ22536 prevented the arteriolar dilations induced by Ex4. Numbers in parentheses indicate the numbers of slices obtained from the same number of animals (biological replicates). Individual data and/or means ± SEM are shown. *p* values—paired *t* test

Next, the effect of GLP-1R activation on cortical arterioles and capillaries was determined in acute brain slices. Vessels in brain slices are lacking tone and are typically fully dilated (due to the absence of the perfusion pressure), therefore, studies of cerebrovascular responses ex vivo require the application of the constricting agents [65]. We used lactate as a constricting agent because the release of this metabolite increases in hypoxic and ischaemic conditions [43, 44, 57]. In a series of preliminary trials, we found that lactate in concentrations of 5 mM and 20 mM in osmolarity-controlled aCSF reduced the diameter of cortical arterioles after 20 min of application by 24 ± 4% and 23 ± 1%, respectively (*p* < 0.001) (Fig. 3a, b). Therefore, in all the subsequent experiments we used 5 mM lactate to constrict the cortical arterioles.

In the presence of 5 mM lactate, bath application of GLP-1R agonist Ex4 (100 nM) caused significant dilations of cortical arterioles (*p* = 0.031), effectively reversing lactate-induced constrictions (Fig. 3a, c). The effect of Ex4 on cortical arterioles was not affected by L-NAME (100 µM) (Fig. 3d), suggesting that GLP-1R-mediated cerebrovascular responses are not mediated by the release and actions of nitric oxide. However, arteriolar dilations induced by Ex4 were prevented by the broad-spectrum adenylate cyclase inhibitor SQ22536 (100 µM) (Fig. 3e), indicating that the vascular effects of GLP-1R activation are mediated by a cAMP/PKA signalling pathway.

**Fig. 4:**
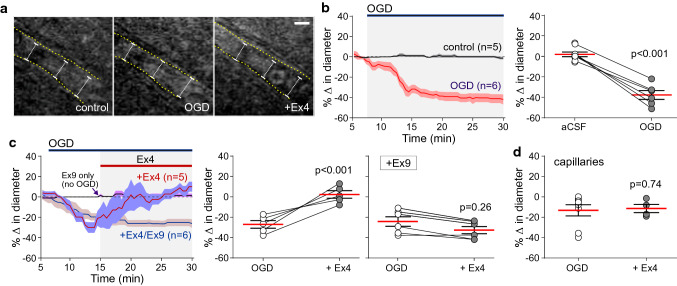
GLP-1 receptor activation dilates cortical arterioles in the ex vivo model of ischaemic stroke. **a** Representative DIC images illustrating a cortical arteriole response to oxygen and glucose deprivation (OGD) followed by GLP-1 receptor agonist Exendin-4 (Ex4; 100 nM) applied in conditions of OGD, recorded in a coronal slice of a rat cerebral cortex. Scale bar = 10 µm. **b** Summary data illustrating changes in the diameter of cortical arterioles recorded over time and peak arteriolar responses induced by OGD. **c** Summary data illustrating the effect of Ex4 on the diameter of cortical arterioles in conditions of OGD in the absence and presence of GLP-1 receptor antagonist Ex9 (1 µM). Arteriolar constrictions induced by OGD were reversed by Ex4. **d** Summary data illustrating peak changes in the diameter of cortical capillaries induced by OGD and after application of Ex4 in conditions of OGD. Numbers in parentheses indicate the numbers of slices obtained from the same number of animals (biological replicates). Individual data and/or means ± SEM are shown. *p* values—paired (**b**, **c**) or unpaired (**d**) *t* test

Next the effect of Ex4 on cerebral blood vessels was investigated in the ex vivo model of ischaemic stroke, induced by oxygen and glucose deprivation of cortical brain slices. Similarly to the effect of lactate, OGD led to strong constrictions of cortical vessels (Fig. 4a, b). OGD reduced cortical capillary diameter by 13 ± 6% (*p* = 0.019) and arteriolar diameter by 27 ± 4% (*p* < 0.001) 10 min after the stimulus onset. Ex4 had no effect on OGD-induced reduction in capillary diameter (*p* = 0.74; Fig. 4d), but reversed the effect of OGD on cortical arterioles (*p* < 0.001; Fig. 4a, c). The dilatory effect of Ex4 on cortical arterioles in conditions of OGD was blocked by Ex9 (1 µM; Fig. 4c), confirming that the effect of Ex4 is specific and mediated by GLP-1Rs.

**Fig. 5:**
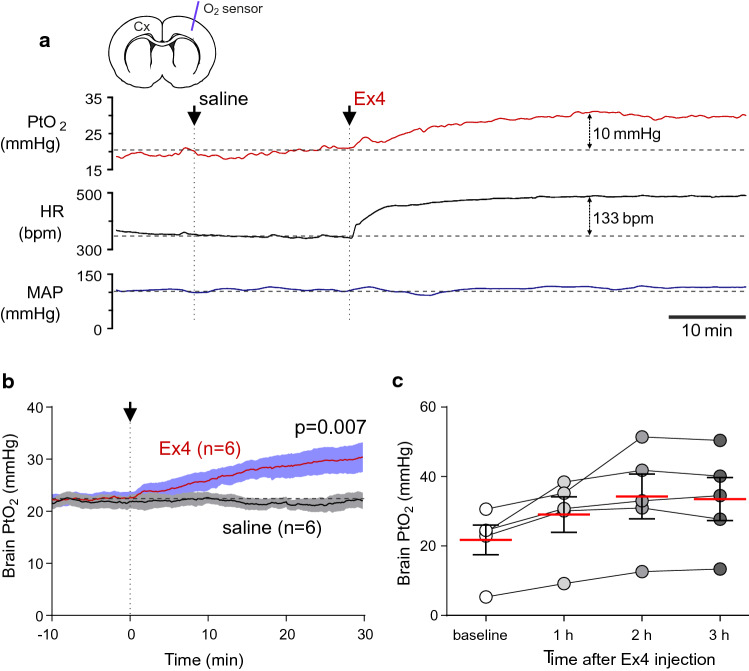
GLP-1 receptor activation increases cerebral blood flow in vivo*.* **a** Representative raw traces of changes in brain tissue partial pressure of oxygen (PtO_2_), heart rate (HR) and mean arterial blood pressure (MAP) after intravenous infusion of saline (0.1 ml) followed by GLP-1 receptor agonist Exendin-4 (Ex4; 10 μg kg^−1^, 0.1 ml volume) in an anaesthetised and artificially ventilated rat. *Cx* cerebral cortex. **b** Summary data illustrating changes in brain PtO_2_ recorded continuously for 30 min after the intravenous infusion of saline or Ex4 (10 μg kg^−1^) in anaesthetised and artificially ventilated rats (*n* = 6). **c** Summary data illustrating the lasting effect of GLP-1R activation on brain PtO_2_ in a separate cohort of animals (*n* = 5). 5 min-long recordings of PtO_2_ were taken at baseline and then 1,2, and 3 h after the administration of Ex4 (10 μg kg^−1^)

To determine the effect of GLP-1R activation on cerebral blood flow in vivo, changes in brain tissue PO_2_ induced by systemic administration of Ex4 (10 μg kg^−1^, intravenously) were recorded in anaesthetised and artificially ventilated rats. It was found that Ex4 induces lasting increases in brain tissue PO_2_ (Fig. 5). Ex4 increased PtO_2_ in the cerebral cortex from 22.0 ± 1.6 mmHg to 30.4 ± 2.9 mmHg (38% increase; *p* = 0.007, *n* = 6) 30 min after the injections, and this effect was sustained for at least 3 h (Fig. 5). Since in this model changes in brain tissue PO_2_ parallel changes in brain perfusion [18], these data indicate that activation of GLP-1Rs increases cerebral blood flow.

## Discussion

Powerful innate mechanisms of inter-organ protection are activated by remote ischaemic conditioning which can be established by cycles of ischaemia/reperfusion applied to an organ/tissue distant from the organ being protected [13]. Numerous experimental studies demonstrated the efficacy of RIC in protecting the brain and the heart against ischaemia/reperfusion injury. We previously proposed that the signalling mechanisms from the remote organ to the heart involve sensory (afferent) innervation of the peripheral tissue undergoing RIC, autonomic parasympathetic innervation of the visceral organs, and the release and actions of GLP-1 [5, 6, 62, 63]. The results of the present study suggest that RIC-induced neuroprotection against ischaemic stroke is mediated by a similar mechanism involving GLP-1R-mediated signalling. This conclusion is supported by the central finding of the present study that the highly selective GLP-1R antagonist Ex9 blocks the neuroprotective effect of RIC.

Several studies reported the neuroprotective effects of GLP-1 and GLP-1R agonists in preclinical models of stroke [9, 21, 45]. Yet, the mechanisms underlying the GLP-1R-mediated neuroprotection are not fully understood. There is evidence that treatment with GLP-1R agonists increases GLP-1R expression, increases the level of the neurotrophic factor BDNF, protects the blood–brain barrier through MMP-9 regulation, decreases microglial activation, oxidative stress and the release of apoptotic factors [41, 48, 50, 51, 78, 85]. Here we tested the hypothesis that GLP-1 actions reduce ischaemic brain tissue damage via activation of GLP-1R on cerebral vasculature leading to the improvement of brain blood flow. The data obtained support this hypothesis by showing that GLP-1R are expressed by the cells lining cortical arterioles and that the GLP-1R agonist Ex4 effectively reverses the constriction of these arterioles induced by lactate or simulated ischaemia (OGD) ex vivo and increases the cerebral blood flow in vivo. Collectively these data suggest that vascular mechanisms are likely to mediate the neuroprotective effects downstream of GLP-1R activation. The importance of preserving microvascular flow in the context of cardioprotection against myocardial ischaemia/reperfusion injury had been recently highlighted [32].

GLP-1Rs have been shown to be expressed by the arterial smooth muscle cells in the peripheral vasculature [71]. The vasodilatory effect of GLP-1 on the aorta was shown to be mediated through glucagon signalling, K_ATP_ channels, PKA phosphorylation [27, 30, 73] and increased NO production [3]. A recent study demonstrated that Ex4 causes dilation of retinal capillaries and this effect is mediated by NO [86]. The data obtained in this study show that the vasodilatory effect of GLP-1R activation on cortical arterioles is mediated by cAMP and is independent of NO production. That cAMP mediates the effects of a GLP-1 analogue is not surprising considering that GLP-1R is a well-characterized G_s_-coupled receptor [17].

In GLP-1R reporter mice, we found that most vascular GLP-1R expression in the cortex is associated with arterioles with no significant receptor expression detected in cells associated with brain capillaries and venules. These data are fully consistent with the results of single-cell RNA sequencing of brain vasculature [80]. Dilation of cortical arterioles induced by GLP-1R activation is likely to lessen the impact of the neurotoxic effects associated with impaired cerebral perfusion in stroke, as early improved perfusion is beneficial for better functional outcomes [28]. Indeed, maintaining/restoring blood supply in the penumbra has been shown to reduce the size and severity of cerebral infarcts [4]. The conclusions of this study are also supported by the recent evidence obtained in rodent models showing a marked reduction of cerebral blood flow after MCAO and improved collateral circulation in response to RIC [46, 55]. However, one of the limitations of our study is that the effect of RIC on collateral circulation was not assessed in conditions of systemic GLP-1R blockade with Ex9.

The source of GLP-1 that acts on GLP-1Rs expressed by cortical arterioles and mediates RIC-induced neuroprotection against ischaemic stroke remains to be determined. In our previously proposed model of RIC-induced cardioprotection, we suggested that cycles of remote ischaemia/reperfusion activate tissue nociceptors [5] that project to the CNS, leading to activation of a specific group of vagal preganglionic neurons [62] that innervate the gut [63], and upon activation stimulate the release of GLP-1 into the systemic circulation, culminating in cardioprotective action of GLP-1 on the heart [6, 81]. It is plausible that the same reflex mechanism underlies RIC-induced neuroprotection: RIC stimulates GLP-1 secretion by enteroendocrine cells of the gut, leading to GLP-1 release into the systemic circulation and its action on the brain vasculature. However, most of the intestinally-derived GLP-1 is believed to be rapidly degraded and low levels of GLP-1 are usually detected in the systemic circulation. For example, in a study showing that GLP-1R activation has protective effects on the endothelium in obese rats and patients, only modest increases in plasma GLP-1 were reported [67]. Yet, there is also evidence that the lasting effects of GLP-1 may persist for some time even when the circulating levels of the hormone return back to the normal level [38].

It is also conceivable that RIC stimulates the release of GLP-1 within the brain by the preproglucagon neurons that reside in the brainstem and have projections to the forebrain [16, 40, 53]. This distinct population of GLP-1-producing neurons reside in the dorsal vagal complex [53] and in close proximity to the pool of vagal preganglionic neurones that are critically important for RIC-induced effects [62]. Conceivably, afferent inputs from the peripheral organ/tissue undergoing RIC activate both neighbouring populations of brainstem neurons leading to simultaneous increases in vagal activity and release of GLP-1 in the CNS. However, the brainstem PPG neurones have no direct projections to the cerebral cortex and GLP-1 receptors are not widely expressed by cortical neurons [16]. Therefore, it seems unlikely that RIC-induced neuroprotection is mediated by direct actions of GLP-1 on brain neurons. Collectively, the data obtained in this study point to the importance of the mechanism that modulates cerebral blood flow and is mediated by vascular GLP-1Rs located on the luminal side of the blood–brain barrier.

Despite strong pre-clinical evidence of the effectiveness of RIC in protecting the heart and the brain against ischaemia/reperfusion injury, it has failed to translate into a clinical treatment of myocardial infarction or stroke. Studies conducted in young and healthy experimental animals reported that RIC can markedly reduce cortical infarcts [10, 72, 84]. Yet, clinical data show no major beneficial effect of RIC on stroke outcomes [68], although with some suggestion of reduced recurrent strokes with intracerebral artery stenosis treatment, and decreased stroke severity with carotid stenosis [69]. We previously demonstrated that RIC is critically dependent on autonomic parasympathetic mechanisms [62] and it is plausible that its clinical efficacy is compromised by the inability of many patients to recruit vagal activity. As we reasoned in our earlier publications [7, 25, 26, 63] and reported supporting evidence [1, 56], vagal tone decreases with age and could be severely diminished or even absent in many disease states, rendering many patients unable to recruit innate mechanisms of inter-organ protection. If GLP-1 is a common mediator of cardio- and neuroprotection induced by RIC, then administration of GLP-1 stable analogues may offer a better therapeutic solution for the treatment of acute myocardial infarction and ischaemic stroke, especially in patients with autonomic dysfunction. Indeed, exenatide (synthetic Ex4) had been shown to reduce the myocardial injury in patients with myocardial infarction [54], although the results of a recent clinical trial studying the individual effects of RIC, exenatide, and their combination (COMBAT-MI trial) showed no effect of these treatments on infarct size [22]. However, as it was highlighted [11] in that study neither the dose given, nor the plasma concentration of exenatide were known to determine the bioavailability of the drug at the time of treatment.

In conclusion, this study shows that GLP-1R activation mediates neuroprotection against ischaemic stroke established by remote ischaemic conditioning. The neuroprotection induced by GLP-1 is likely to be mediated via its action on cortical arterioles and improved perfusion in the areas surrounding the infarcted brain tissue.

## References

[1] Ackland GL, Whittle J, Toner A, Machhada A, Del Arroyo AG, Sciuso A, Jenkins N, Dyson A, Struthers R, Sneyd JR, Minto G, Singer M, Shah AM, Gourine AV (2016). Molecular mechanisms linking autonomic dysfunction and impaired cardiac contractility in critical illness. Crit Care Med.

[2] Ang R, Mastitskaya S, Hosford PS, Basalay M, Specterman M, Aziz Q, Li Y, Orini M, Taggart P, Lambiase PD, Gourine A, Tinker A, Gourine AV (2018). Modulation of cardiac ventricular excitability by GLP-1 (glucagon-like peptide-1). Circ Arrhythm Electrophysiol.

[3] Ban K, Noyan-Ashraf MH, Hoefer J, Bolz SS, Drucker DJ, Husain M (2008). Cardioprotective and vasodilatory actions of glucagon-like peptide 1 receptor are mediated through both glucagon-like peptide 1 receptor-dependent and -independent pathways. Circulation.

[4] Baron JC (2018). Protecting the ischaemic penumbra as an adjunct to thrombectomy for acute stroke. Nat Rev Neurol.

[5] Basalay M, Barsukevich V, Mastitskaya S, Mrochek A, Pernow J, Sjöquist P-O, Ackland GL, Gourine AV, Gourine A (2012). Remote ischaemic pre- and delayed postconditioning—similar degree of cardioprotection but distinct mechanisms. Exp Physiol.

[6] Basalay MV, Mastitskaya S, Mrochek A, Ackland GL, Del Arroyo AG, Sanchez J, Sjoquist P-O, Pernow J, Gourine AV, Gourine A (2016). Glucagon-like peptide-1 (GLP-1) mediates cardioprotection by remote ischaemic conditioning. Cardiovasc Res.

[7] Basalay M, Mastitskaya S, Mrochek A, Ackland GL, del Arroyo AG, Sanchez J, Sjoquist P-O, Pernow J, Gourine AV, Gourine A (2017). Reply: Glucagon-like peptide-1 mediates cardioprotection by remote ischaemic conditioning. Cardiovasc Res.

[8] Basalay MV, Davidson SM, Gourine AV, Yellon DM (2018). Neural mechanisms in remote ischaemic conditioning in the heart and brain: Mechanistic and translational aspects. Basic Res Cardiol.

[9] Basalay MV, Davidson SM, Yellon DM (2019). Neuroprotection in rats following ischaemia-reperfusion injury by GLP-1 analogues—liraglutide and semaglutide. Cardiovasc Drugs Ther.

[10] Basalay MV, Wiart M, Chauveau F, Dumot C, Leon C, Amaz C, Bolbos R, Cash D, Kim E, Mechtouff L, Cho TH, Nighoghossian N, Davidson SM, Ovize M, Yellon DM (2020). Neuroprotection by remote ischemic conditioning in the setting of acute ischemic stroke: a preclinical two-centre study. Sci Rep.

[11] Bøtker HE (2021). Searching myocardial rescue through intermittent upper arm occlusion and lizard saliva. Basic Res Cardiol.

[12] Brandli A (2015). Remote limb ischemic preconditioning: a neuroprotective technique in rodents. J Vis Exp.

[13] Bromage DI, Pickard JMJ, Rossello X, Ziff OJ, Burke N, Yellon DM, Davidson SM (2017). Remote ischaemic conditioning reduces infarct size in animal in vivo models of ischaemia-reperfusion injury: a systematic review and meta-analysis. Cardiovasc Res.

[14] Chen G, Thakkar M, Robinson C, Doré S (2018). Limb remote ischemic conditioning: mechanisms, anesthetics, and the potential for expanding therapeutic options. Front Neurol.

[15] Cheng X, Zhao H, Yan F, Tao Z, Wang R, Han Z, Li G, Luo Y, Ji X (2018). Limb remote ischemic post-conditioning mitigates brain recovery in a mouse model of ischemic stroke by regulating reactive astrocytic plasticity. Brain Res.

[16] Cork SC, Richards JE, Holt MK, Gribble FM, Reimann F, Trapp S (2015). Distribution and characterisation of Glucagon-like peptide-1 receptor expressing cells in the mouse brain. Mol Metab.

[17] de Graaf C, Donnelly D, Wootten D, Lau J, Sexton PM, Miller LJ, Ahn J-M, Liao J, Fletcher MM, Yang D, Brown AJH, Zhou C, Deng J, Wang M-W (2016). Glucagon-like peptide-1 and its class B G protein-coupled receptors: a long march to therapeutic successes. Pharmacol Rev.

[18] Demchenko IT, Luchakov YI, Moskvin AN, Gutsaeva DR, Allen BW, Thalmann ED, Piantadosi CA (2005). Cerebral blood flow and brain oxygenation in rats breathing oxygen under pressure. J Cereb Blood Flow Metab.

[19] During MJ, Cao L, Zuzga DS, Francis JS, Fitzsimons HL, Jiao X, Bland RJ, Klugmann M, Banks WA, Drucker DJ, Haile CN (2003). Glucagon-like peptide-1 receptor is involved in learning and neuroprotection. Nat Med.

[20] England TJ, Hedstrom A, O’Sullivan S, Donnelly R, Barrett DA, Sarmad S, Sprigg N, Bath PM (2017). RECAST (remote ischemic conditioning after stroke trial): a pilot randomized placebo controlled phase II trial in acute ischemic stroke. Stroke.

[21] Erbil D, Eren CY, Demirel C, Küçüker MU, Solaroğlu I, Eser HY (2019). GLP-1’s role in neuroprotection: a systematic review. Brain Inj.

[22] García Del Blanco B, Otaegui I, Rodríguez-Palomares JF, Bayés-Genis A, Fernández-Nofrerías E, Vilalta Del Olmo V, Carrillo X, Ibáñez B, Worner F, Casanova J, Pueo E, González-Juanatey JR, López-Pais J, Bardají A, Bonet G, Fuertes M, Rodríguez-Sinovas A, Ruiz-Meana M, Inserte J, Barba I, Gómez-Talavera S, Martí G, Serra B, Bellera N, Ojeda-Ramos M, Cuellar H, Valente F, Carmona MÁ, Miró-Casas E, Marsal JR, Sambola A, Lidón RM, Bañeras J, Elízaga J, Padilla F, Barrabés JA, Hausenloy DJ, Ferreira-González I, García-Dorado D (2021). Effect of COMBinAtion therapy with remote ischemic conditioning and exenatide on the Myocardial Infarct size: a two-by-two factorial randomized trial (COMBAT-MI). Basic Res Cardiol.

[23] Gaspar A, Lourenço AP, Pereira MÁ, Azevedo P, Roncon-Albuquerque R, Marques J, Leite-Moreira AF (2018). Randomized controlled trial of remote ischaemic conditioning in ST-elevation myocardial infarction as adjuvant to primary angioplasty (RIC-STEMI). Basic Res Cardiol.

[24] Gedik N, Kottenberg E, Thielmann M, Frey UH, Jakob H, Peters J, Heusch G, Kleinbongard P (2017). Potential humoral mediators of remote ischemic preconditioning in patients undergoing surgical coronary revascularization. Sci Rep.

[25] Gourine AV, Ackland GL (2018). Cardiac vagus and exercise. Physiology.

[26] Gourine A, Gourine AV (2014). Neural mechanisms of cardioprotection. Physiology.

[27] Green BD, Hand KV, Dougan JE, McDonnell BM, Cassidy RS, Grieve DJ (2008). GLP-1 and related peptides cause concentration-dependent relaxation of rat aorta through a pathway involving KATP and cAMP. Arch Biochem Biophys.

[28] Gregori-Pla C, Blanco I, Camps-Renom P, Zirak P, Serra I, Cotta G, Maruccia F, Prats-Sánchez L, Martínez-Domeño A, Busch DR, Giacalone G, Martí-Fàbregas J, Durduran T, Delgado-Mederos R (2019). Early microvascular cerebral blood flow response to head-of-bed elevation is related to outcome in acute ischemic stroke. J Neurol.

[29] Hahn CD, Manlhiot C, Schmidt MR, Nielsen TT, Redington AN (2011). Remote ischemic per-conditioning: a novel therapy for acute stroke?. Stroke.

[30] Han F, Hou N, Liu Y, Huang N, Pan R, Zhang X, Mao E, Sun X (2019). Liraglutide improves vascular dysfunction by regulating a cAMP-independent PKA-AMPK pathway in perivascular adipose tissue in obese mice. Biomed Pharmacother.

[31] Hess DC, Blauenfeldt RA, Andersen G, Hougaard KD, Hoda MN, Ding Y, Ji X (2015). Remote ischaemic conditioning-a new paradigm of self-protection in the brain. Nat Rev Neurol.

[32] Heusch G (2019). Coronary microvascular obstruction: the new frontier in cardioprotection. Basic Res Cardiol.

[33] Heusch G (2020). Myocardial ischaemia–reperfusion injury and cardioprotection in perspective. Nat Rev Cardiol.

[34] Holst JJ (2007). The physiology of glucagon-like peptide 1. Physiol Rev.

[35] Holt MK, Richards JE, Cook DR, Brierley DI, Williams DL, Reimann F, Gribble FM, Trapp S (2019). Preproglucagon neurons in the nucleus of the solitary tract are the main source of brain GLP-1, mediate stress-induced hypophagia, and limit unusually large intakes of food. Diabetes.

[36] Holt MK, Cook DR, Brierley DI, Richards JE, Reimann F, Gourine AV, Marina N, Trapp S (2020). PPG neurons in the nucleus of the solitary tract modulate heart rate but do not mediate GLP-1 receptor agonist-induced tachycardia in mice. Mol Metab.

[37] Hougaard KD, Hjort N, Zeidler D, Sørensen L, Nørgaard A, Hansen TM, von Weitzel-Mudersbach P, Simonsen CZ, Damgaard D, Gottrup H, Svendsen K, Rasmussen PV, Ribe LR, Mikkelsen IK, Nagenthiraja K, Cho T-H, Redington AN, Bøtker HE, Østergaard L, Mouridsen K, Andersen G (2014). Remote ischemic perconditioning as an adjunct therapy to thrombolysis in patients with acute ischemic stroke: a randomized trial. Stroke.

[38] Hui H, Farilla L, Merkel P, Perfetti R (2002). The short half-life of glucagon-like peptide-1 in plasma does not reflect its long-lasting beneficial effects. Eur J Endocrinol.

[39] Ibáñez B, Heusch G, Ovize M, Van de Werf F (2015). Evolving therapies for myocardial ischemia/reperfusion injury. J Am Coll Cardiol.

[40] Jin S-LC, Han VKM, Simmons JG, Towle AC, Lauder JM, Lund PK (1988). Distribution of glucagonlike peptide I (GLP-I), glucagon, and glicentin in the rat brain: an immunocytochemical study. J Comp Neurol.

[41] Jin J, Kang HM, Jung J, Jeong JW, Park C (2014). Related expressional change of HIF-1α to the neuroprotective activity of exendin-4 in transient global ischemia. NeuroReport.

[42] Johnson CO, Nguyen M, Roth GA, Nichols E, Alam T, Abate D, Abd-Allah F, Abdelalim A, Abraha HN, Abu-Rmeileh NM, Adebayo OM, Adeoye AM, Agarwal G, Agrawal S, Aichour AN, Aichour I, Aichour MTE, Alahdab F, Ali R, Alvis-Guzman N, Anber NH, Anjomshoa M, Arabloo J, Arauz A, Ärnlöv J, Arora A, Awasthi A, Banach M, Barboza MA, Barker-Collo SL, Bärnighausen TW, Basu S, Belachew AB, Belayneh YM, Bennett DA, Bensenor IM, Bhattacharyya K, Biadgo B, Bijani A, Bikbov B, Bin Sayeed MS, Butt ZA, Cahuana-Hurtado L, Carrero JJ, Carvalho F, Castañeda-Orjuela CA, Castro F, Catalá-López F, Chaiah Y, Chiang PPC, Choi JYJ, Christensen H, Chu DT, Cortinovis M, Damasceno AAM, Dandona L, Dandona R, Daryani A, Davletov K, De Courten B, De la Cruz-Góngora V, Degefa MG, Dharmaratne SD, Diaz D, Dubey M, Duken EE, Edessa D, Endres M, Faraon EJA, Farzadfar F, Fernandes E, Fischer F, Flor LS, Ganji M, Gebre AK, Gebremichael TG, Geta B, Gezae KE, Gill PS, Gnedovskaya EV, Gómez-Dantés H, Goulart AC, Grosso G, Guo Y, Gupta R, Haj-Mirzaian A, Haj-Mirzaian A, Hamidi S, Hankey GJ, Hassen HY, Hay SI, Hegazy MI, Heidari B, Herial NA, Hosseini MA, Hostiuc S, Irvani SSN, Islam SMS, Jahanmehr N, Javanbakht M, Jha RP, Jonas JB, Józwiak JJ, Jürisson M, Kahsay A, Kalani R, Kalkonde Y, Kamil TA, Kanchan T, Karch A, Karimi N, Karimi-Sari H, Kasaeian A, Kassa TD, Kazemeini H, Kefale AT, Khader YS, Khalil IA, Khan EA, Khang YH, Khubchandani J, Kim D, Kim YJ, Kisa A, Kivimäki M, Koyanagi A, Krishnamurthi RK, Anil Kumar G, Lafranconi A, Lewington S, Li S, Lo WD, Lopez AD, Lorkowski S, Lotufo PA, Mackay MT, Majdan M, Majdzadeh R, Majeed A, Malekzadeh R, Manafi N, Mansournia MA, Mehndiratta MM, Mehta V, Mengistu G, Meretoja A, Meretoja TJ, Miazgowski B, Miazgowski T, Miller TR, Mirrakhimov EM, Mohajer B, Mohammad Y, Mohammadoo-Khorasani M, Mohammed S, Mohebi F, Mokdad AH, Mokhayeri Y, Moradi G, Morawska L, Moreno Velásquez I, Mousavi SM, Muhammed OSS, Muruet W, Naderi M, Naghavi M, Naik G, Nascimento BR, Negoi RI, Nguyen CT, Nguyen LH, Nirayo YL, Norrving B, Noubiap JJ, Ofori-Asenso R, Ogbo FA, Olagunju AT, Olagunju TO, Owolabi MO, Pandian JD, Patel S, Perico N, Piradov MA, Polinder S, Postma MJ, Poustchi H, Prakash V, Qorbani M, Rafiei A, Rahim F, Rahimi K, Rahimi-Movaghar V, Rahman M, Rahman MA, Reis C, Remuzzi G, Renzaho AMN, Ricci S, Roberts NLS, Robinson SR, Roever L, Roshandel G, Sabbagh P, Safari H, Safari S, Safiri S, Sahebkar A, Salehi Zahabi S, Samy AM, Santalucia P, Santos IS, Santos JV, Santric Milicevic MM, Sartorius B, Sawant AR, Schutte AE, Sepanlou SG, Shafieesabet A, Shaikh MA, Shams-Beyranvand M, Sheikh A, Sheth KN, Shibuya K, Shigematsu M, Shin MJ, Shiue I, Siabani S, Sobaih BH, Sposato LA, Sutradhar I, Sylaja PA, Szoeke CEI, Te Ao BJ, Temsah MH, Temsah O, Thrift AG, Tonelli M, Topor-Madry R, Tran BX, Tran KB, Truelsen TC, Tsadik AG, Ullah I, Uthman OA, Vaduganathan M, Valdez PR, Vasankari TJ, Vasanthan R, Venketasubramanian N, Vosoughi K, Vu GT, Waheed Y, Weiderpass E, Weldegwergs KG, Westerman R, Wolfe CDA, Wondafrash DZ, Xu G, Yadollahpour A, Yamada T, Yatsuya H, Yimer EM, Yonemoto N, Yousefifard M, Yu C, Zaidi Z, Zamani M, Zarghi A, Zhang Y, Zodpey S, Feigin VL, Vos T, Murray CJL (2019). Global, regional, and national burden of stroke, 1990–2016: a systematic analysis for the Global Burden of Disease Study 2016. Lancet Neurol.

[43] Karagiannis A, Sylantyev S, Hadjihambi A, Hosford PS, Kasparov S, Gourine AV (2016). Hemichannel-mediated release of lactate. J Cereb Blood Flow Metab.

[44] Karaszewski B, Wardlaw JM, Marshall I, Cvoro V, Wartolowska K, Haga K, Armitage PA, Bastin ME, Dennis MS (2009). Early brain temperature elevation and anaerobic metabolism in human acute ischaemic stroke. Brain.

[45] Kerendi F, Kin H, Halkos ME, Jiang R, Zatta AJ, Zhao ZQ, Guyton RA, Vinten-Johansen J (2005). Remote postconditioning: Brief renal ischemia and reperfusion applied before coronary artery reperfusion reduces myocardial infarct size via endogenous activation of adenosine receptors. Basic Res Cardiol.

[46] Kitagawa K, Saitoh M, Ishizuka K, Shimizu S (2018). Remote limb ischemic conditioning during cerebral ischemia reduces infarct size through enhanced collateral circulation in murine focal cerebral ischemia. J Stroke Cerebrovasc Dis.

[47] Kleinbongard P, Skyschally A, Heusch G (2017). Cardioprotection by remote ischemic conditioning and its signal transduction. Pflugers Arch.

[48] Kuroki T, Tanaka R, Shimada Y, Yamashiro K, Ueno Y, Shimura H, Urabe T, Hattori N (2016). Exendin-4 inhibits matrix metalloproteinase-9 activation and reduces infarct growth after focal cerebral ischemia in hyperglycemic mice. Stroke.

[49] Landman TRJ, Schoon Y, Warlé MC, de Leeuw F-E, Thijssen DHJ (2019). Remote ischemic conditioning as an additional treatment for acute ischemic stroke. Stroke.

[50] Lee CH, Yan B, Yoo K-Y, Choi JH, Kwon S-H, Her S, Sohn Y, Hwang IK, Cho JH, Kim Y-M, Won M-H (2011). Ischemia-induced changes in glucagon-like peptide-1 receptor and neuroprotective effect of its agonist, exendin-4, in experimental transient cerebral ischemia. J Neurosci Res.

[51] Li Y, Perry TA, Kindy MS, Harvey BK, Tweedie D, Holloway HW, Powers K, Shen H, Egan JM, Sambamurti K, Brossi A, Lahiri DK, Mattson MP, Hoffer BJ, Wang Y, Greig NH (2009). GLP-1 receptor stimulation preserves primary cortical and dopaminergic neurons in cellular and rodent models of stroke and Parkinsonism. Proc Natl Acad Sci USA.

[52] Lieder HR, Kleinbongard P, Skyschally A, Hagelschuer H, Chilian WM, Heusch G (2018). Vago-splenic axis in signal transduction of remote ischemic preconditioning in pigs and rats. Circ Res.

[53] Llewellyn-Smith IJ, Reimann F, Gribble FM, Trapp S (2011). Preproglucagon neurons project widely to autonomic control areas in the mouse brain. Neuroscience.

[54] Lønborg J, Kelbæk H, Vejlstrup N, Bøtker HE, Kim WY, Holmvang L, Jørgensen E, Helqvist S, Saunamäki K, Terkelsen CJ, Schoos MM, Køber L, Clemmensen P, Treiman M, Engstrøm T (2012). Exenatide reduces final infarct size in patients with ST-segment-elevation myocardial infarction and short-duration of ischemia. Circ Cardiovasc Interv.

[55] Ma J, Ma Y, Shuaib A, Winship IR (2020). Improved collateral flow and reduced damage after remote ischemic perconditioning during distal middle cerebral artery occlusion in aged rats. Sci Rep.

[56] Machhada A, Trapp S, Marina N, Stephens RCM, Whittle J, Lythgoe MF, Kasparov S, Ackland GL, Gourine AV (2017). Vagal determinants of exercise capacity. Nat Commun.

[57] Magistretti PJ, Allaman I (2018). Lactate in the brain: From metabolic end-product to signalling molecule. Nat Rev Neurosci.

[58] Marina N, Christie IN, Korsak A, Doronin M, Brazhe A, Hosford PS, Wells JA, Sheikhbahaei S, Humoud I, Paton JFR, Lythgoe MF, Semyanov A, Kasparov S, Gourine AV (2020). Astrocytes monitor cerebral perfusion and control systemic circulation to maintain brain blood flow. Nat Commun.

[59] Marlet IR, Ölmestig JNE, Vilsbøll T, Rungby J, Kruuse C (2018). Neuroprotective mechanisms of glucagon-like peptide-1-based therapies in ischaemic stroke: a systematic review based on pre-clinical studies. Basic Clin Pharmacol Toxicol.

[60] Marso SP, Daniels GH, Brown-Frandsen K, Kristensen P, Mann JFE, Nauck MA, Nissen SE, Pocock S, Poulter NR, Ravn LS, Steinberg WM, Stockner M, Zinman B, Bergenstal RM, Buse JB, Steering Committee LEADER, Trial Investigators LEADER (2016). Liraglutide and cardiovascular outcomes in type 2 diabetes. N Engl J Med.

[61] Marso SP, Bain SC, Consoli A, Eliaschewitz FG, Jódar E, Leiter LA, Lingvay I, Rosenstock J, Seufert J, Warren ML, Woo V, Hansen O, Holst AG, Pettersson J, Vilsbøll T, SUSTAIN-6 Investigators (2016). Semaglutide and cardiovascular outcomes in patients with type 2 diabetes. N Engl J Med.

[62] Mastitskaya S, Marina N, Gourine A, Gilbey MP, Spyer KM, Teschemacher AG, Kasparov S, Trapp S, Ackland GL, Gourine AV (2012). Cardioprotection evoked by remote ischaemic preconditioning is critically dependent on the activity of vagal pre-ganglionic neurones. Cardiovasc Res.

[63] Mastitskaya S, Basalay M, Hosford PS, Ramage AG, Gourine A, Gourine AV (2016). Identifying the source of a humoral factor of remote (pre)conditioning cardioprotection. PLoS ONE.

[64] Mishra A, O’Farrell FM, Reynell C, Hamilton NB, Hall CN, Attwell D (2014). Imaging pericytes and capillary diameter in brain slices and isolated retinae. Nat Protoc.

[65] Mishra A, Reynolds JP, Chen Y, Gourine AV, Rusakov DA, Attwell D (2016). Astrocytes mediate neurovascular signaling to capillary pericytes but not to arterioles. Nat Neurosci.

[66] Müller TD, Finan B, Bloom SR, D’Alessio D, Drucker DJ, Flatt PR, Fritsche A, Gribble F, Grill HJ, Habener JF, Holst JJ, Langhans W, Meier JJ, Nauck MA, Perez-Tilve D, Pocai A, Reimann F, Sandoval DA, Schwartz TW, Seeley RJ, Stemmer K, Tang-Christensen M, Woods SC, DiMarchi RD, Tschöp MH (2019). Glucagon-like peptide 1 (GLP-1). Mol Metab.

[67] Osto E, Doytcheva P, Corteville C, Bueter M, Dörig C, Stivala S, Buhmann H, Colin S, Rohrer L, Hasballa R, Tailleux A, Wolfrum C, Tona F, Manz J, Vetter D, Spliethoff K, Vanhoutte PM, Landmesser U, Pattou F, Staels B, Matter CM, Lutz TA, Lüscher TF (2015). Rapid and body weight-independent improvement of endothelial and high-density lipoprotein function after Roux-en-Y gastric bypass: role of glucagon-like peptide-1. Circulation.

[68] Pico F, Lapergue B, Ferrigno M, Rosso C, Meseguer E, Chadenat ML, Bourdain F, Obadia M, Hirel C, Duong DL, Deltour S, Aegerter P, Labreuche J, Cattenoy A, Smadja D, Hosseini H, Guillon B, Wolff V, Samson Y, Cordonnier C, Amarenco P (2020). Effect of in-hospital remote ischemic perconditioning on brain infarction growth and clinical outcomes in patients with acute ischemic Stroke: The RESCUE BRAIN randomized clinical trial. JAMA Neurol.

[69] Purroy F, García C, Mauri G, Pereira C, Torres C, Vazquez-Justes D, Vicente-Pascual M, Vena A, Arque G (2020). Induced neuroprotection by remote ischemic perconditioning as a new paradigm in ischemic stroke at the acute phase, a systematic review. BMC Neurol.

[70] Qin C, Yan X, Jin H, Zhang R, He Y, Sun X, Zhang Y, Guo Z-N, Yang Y (2020). Effects of remote ischemic conditioning on cerebral hemodynamics in ischemic stroke. Neuropsychiatr Dis Treat.

[71] Richards P, Parker HE, Adriaenssens AE, Hodgson JM, Cork SC, Trapp S, Gribble FM, Reimann F (2014). Identification and characterization of GLP-1 receptor-expressing cells using a new transgenic mouse model. Diabetes.

[72] Ripley AJ, Jeffers MS, McDonald MW, Montroy J, Dykes A, Fergusson DA, Silasi G, Lalu MM, Corbett D (2021). Neuroprotection by remote ischemic conditioning in rodent models of focal ischemia: a systematic review and meta-analysis. Transl Stroke Res.

[73] Sélley E, Kun S, Szijártó IA, Kertész M, Wittmann I, Molnár GA (2016). Vasodilator effect of glucagon: receptorial crosstalk among glucagon, GLP-1, and receptor for glucagon and GLP-1. Horm Metab Res.

[74] Seners P, Turc G, Maïer B, Mas J-L, Oppenheim C, Baron J-C (2016). Incidence and predictors of early recanalization after intravenous thrombolysis: a systematic review and meta-analysis. Stroke.

[75] Skyschally A, Gent S, Amanakis G, Schulte C, Kleinbongard P, Heusch G (2015). Across-species transfer of protection by remote ischemic preconditioning with species-specific myocardial signal transduction by reperfusion injury salvage kinase and survival activating factor enhancement pathways. Circ Res.

[76] Spratt NJ, Fernandez J, Chen M, Rewell S, Cox S, van Raay L, Hogan L, Howells DW (2006). Modification of the method of thread manufacture improves stroke induction rate and reduces mortality after thread-occlusion of the middle cerebral artery in young or aged rats. J Neurosci Methods.

[77] Sun J, Tong L, Luan Q, Deng J, Li Y, Li Z, Dong H, Xiong L (2012). Protective effect of delayed remote limb ischemic postconditioning: role of mitochondrial K(ATP) channels in a rat model of focal cerebral ischemic reperfusion injury. J Cereb Blood Flow Metab.

[78] Teramoto S, Miyamoto N, Yatomi K, Tanaka Y, Oishi H, Arai H, Hattori N, Urabe T (2011). Exendin-4, a glucagon-like peptide-1 receptor agonist, provides neuroprotection in mice transient focal cerebral ischemia. J Cereb Blood Flow Metab.

[79] Van Der Zijden JP, Bouts MJRJ, Wu O, Roeling TAP, Bleys RLAW, Van Der Toorn A, Dijkhuizen RM (2008). Manganese-enhanced MRI of brain plasticity in relation to functional recovery after experimental stroke. J Cereb Blood Flow Metab.

[80] Vanlandewijck M, He L, Mäe MA, Andrae J, Ando K, Del Gaudio F, Nahar K, Lebouvier T, Laviña B, Gouveia L, Sun Y, Raschperger E, Räsänen M, Zarb Y, Mochizuki N, Keller A, Lendahl U, Betsholtz C (2018). A molecular atlas of cell types and zonation in the brain vasculature. Nature.

[81] Verouhis D, Saleh N, Settergren M, Sörensson P, Gourine A, Pernow J (2019). Remote ischemic conditioning protects against endothelial ischemia-reperfusion injury via a glucagon-like peptide-1 receptor-mediated mechanism in humans. Int J Cardiol.

[82] Vinciguerra A, Cepparulo P, Anzilotti S, Cuomo O, Valsecchi V, Amoroso S, Annunziato L, Pignataro G (2020). Remote postconditioning ameliorates stroke damage by preventing let-7a and miR-143 up-regulation. Theranostics.

[83] Wang Y, Meng R, Song H, Liu G, Hua Y, Cui D, Zheng L, Feng W, Liebeskind DS, Fisher M, Ji X (2017). Remote ischemic conditioning may improve outcomes of patients with cerebral small-vessel disease. Stroke.

[84] Weir P, Maguire R, O’Sullivan SE, England TJ (2020). A meta-analysis of remote ischaemic conditioning in experimental stroke. J Cereb Blood Flow Metab.

[85] Yang D, Nakajo Y, Iihara K, Kataoka H, Yanamoto H (2013). Alogliptin, a dipeptidylpeptidase-4 inhibitor, for patients with diabetes mellitus type 2, induces tolerance to focal cerebral ischemia in non-diabetic, normal mice. Brain Res.

[86] Zhai R, Xu H, Hu F, Wu J, Kong X, Sun X (2020). GLP-1 receptor agonist exendin-4 regulates retinal capillary tone and restores microvascular patency under ischemia-reperfusion injury. Br J Pharmacol.

